# Genome Sequence of *Salmonella* Phage 9NA

**DOI:** 10.1128/genomeA.00531-14

**Published:** 2014-08-21

**Authors:** Sherwood R. Casjens, Justin C. Leavitt, Graham F. Hatfull, Roger W. Hendrix

**Affiliations:** a Division of Microbiology and Immunology, Pathology Department, University of Utah School of Medicine, Salt Lake City, Utah, USA; b Biology Department, University of Utah, Salt Lake City, Utah, USA; c Department of Biological Sciences, University of Pittsburgh, Pittsburgh, Pennsylvania, USA

## Abstract

The virulent double-stranded DNA (dsDNA) bacteriophage 9NA infects *Salmonella enterica* serovar Typhimurium and has a long noncontractile tail. We report its complete 52,869-bp genome sequence. Phage 9NA and two closely related *S. enterica* serovar Newport phages represent a tailed phage type whose molecular lifestyle has not yet been studied in detail.

## GENOME ANNOUNCEMENT

Only a very small fraction of extant phages have been characterized, and as part of an effort to delineate the varieties of tailed phages that infect *Salmonella* bacteria, we determined the nucleotide sequence of the genome of *Salmonella enterica* serovar Typhimurium phage 9NA. Its virion has typical *Siphoviridae* morphology, with an isometric head and long noncontractile tail that has a brushy baseplate at its tip (1, 2).

Phage 9NA was propagated on *S. enterica* serovar Typhimurium strain LT2, and its DNA was isolated and sequenced by Sanger sequencing, with 9-fold coverage and an average read length of 660 bp (3). Its genome is 52,869 bp long, with 42.9% G+C content. Assembly of the 9ΝΑ sequence resulted in a single circular contig, which suggests that its linear virion DNA is either circularly permuted or has specific direct terminal repeats (4). We have shown experimentally that after cleavage with restriction enzymes, its virion DNA shows a *pac* fragment and no other specific end fragments, which strongly suggests that it utilizes a *pac* site headful DNA packaging mechanism (S. R. Casjens, unpublished data). We predict 84 genes in the 9NA genome; 29 are unique to this phage group, and functions were predicted for only 17 genes. Those 17 genes include DNA metabolism genes and virion assembly genes that have rather distant homologies to known proteins; the major capsid and tail shaft proteins were identified by N-terminal amino acid sequencing (S. R. Casjens, unpublished data). These virion assembly genes lie in the canonical order for medium-sized tailed phages (5, 6); however, an unusually large number of small novel genes are interspersed among them. No genes indicative of a lysogenic lifestyle were identified, indicating that 9NA is a virulent phage, a notion that is supported by its clear plaques. While this work was under way, the incomplete sequences of two *S. enterica* serovar Newport phages, FSL_SP-062 and FSL_SP-069, were reported that are close relatives of 9NA (7).

Like its homologues from *Salmonella* phages P22 and Det7, the 9NA tailspike protein binds to the *S*. Typhimurium O antigen and has endorhamnosidase activity that cleaves this polysaccharide receptor (2). Its gene lies in the opposite orientation and downstream of the other 9NA virion assembly genes, and it is quite similar to the tailspikes of a number of other *S*. Typhimurium phages (2, 8). This protein domain is known to have undergone recent and frequent horizontal exchanges among *S*. Typhimurium tailed phages (8). Other than the tailspike gene, phage 9NA genes have no close relatives to phages whose genomes have been completely sequenced. For example, major capsid protein, tail tip protein, and helicase are only about 27%, 29%, and 39% identical to their closest known relatives, respectively (which are encoded by *Roseobacter* phage RDKLϕ1*, Pseudomonas* phage KPP10, and *Edwardsiella* phage MSW-3, respectively). Phage 9NA and the two FSL phages (above) all carry syntenic and rather similar virion assembly and DNA metabolism genes, but like members of other tailed phage groups, their genomes are mosaically related to one another. They clearly exemplify a tailed phage type whose lifestyle has not yet been studied in molecular detail.

### Nucleotide sequence accession number.

The complete genome sequence of phage 9NA is available in GenBank under the accession no. KJ802832.
